# Levistilide A Exerts a Neuroprotective Effect by Suppressing Glucose Metabolism Reprogramming and Preventing Microglia Polarization Shift: Implications for Parkinson’s Disease

**DOI:** 10.3390/molecules29040912

**Published:** 2024-02-19

**Authors:** Mingjie Zhang, Congyan Duan, Weifang Lin, Honghua Wu, Lu Chen, Hong Guo, Minyu Yu, Qi Liu, Yaling Nie, Hong Wang, Shaoxia Wang

**Affiliations:** 1 School of Medical Technology, Tianjin University of Traditional Chinese Medicine, 10 Poyanghu Road, West Area, Tuanbo New Town, Jinghai District, Tianjin 301617, China; arisaema1307@163.com (M.Z.); duancy19990514@163.com (C.D.); 17608484251@163.com (W.L.); yumy@tjutcm.edu.cn (M.Y.); liuqi23@tjutcm.edu.cn (Q.L.); 17783176851@163.com (Y.N.); 2 State Key Laboratory of Component-Based Chinese Medicine, Tianjin University of Traditional Chinese Medicine, 10 Poyanghu Road, West Area, Tuanbo New Town, Jinghai District, Tianjin 301617, China; wuhonghua2011@tjutcm.edu.cn (H.W.); chenlutjutcm@tjutcm.edu.cn (L.C.); cacti1983@163.com (H.G.)

**Keywords:** Levistilide A, microglia, metabolic reprogramming, Parkinson’s disease

## Abstract

The microglia, displaying diverse phenotypes, play a significant regulatory role in the development, progression, and prognosis of Parkinson’s disease. Research has established that glycolytic reprogramming serves as a critical regulator of inflammation initiation in pro-inflammatory macrophages. Furthermore, the modulation of glycolytic reprogramming has the potential to reverse the polarized state of these macrophages. Previous studies have shown that Levistilide A (LA), a phthalide component derived from Angelica sinensis, possesses a range of pharmacological effects, including anti-inflammatory, antioxidant, and neuroprotective properties. In our study, we have examined the impact of LA on inflammatory cytokines and glucose metabolism in microglia induced by lipopolysaccharide (LPS). Furthermore, we explored the effects of LA on the AMPK/mTOR pathway and assessed its neuroprotective potential both in vitro and in vivo. The findings revealed that LA notably diminished the expression of M1 pro-inflammatory factors induced by LPS in microglia, while leaving M2 anti-inflammatory factor expression unaltered. Additionally, it reduced ROS production and suppressed IκB-α phosphorylation levels as well as NF-κB p65 nuclear translocation. Notably, LA exhibited the ability to reverse microglial glucose metabolism reprogramming and modulate the phosphorylation levels of AMPK/mTOR. In vivo experiments further corroborated these findings, demonstrating that LA mitigated the death of TH-positive dopaminergic neurons and reduced microglia activation in the ventral SNpc brain region of the midbrain and the striatum. In summary, LA exhibited neuroprotective benefits by modulating the polarization state of microglia and altering glucose metabolism, highlighting its therapeutic potential.

## 1. Introduction

Parkinson’s disease (PD) is a common neurodegenerative condition that predominantly affects individuals over the age of 65 and becomes increasingly prevalent with advancing age. According to the World Health Organization, the global burden of PD-related disability and mortality is escalating at a faster rate than any other neurological condition. Currently, levodopa serves as the primary clinical treatment for PD, alleviating its signs and symptoms. However, long-term use of levodopa can lead to motor disorders and a decline in its efficacy [1]. Therefore, the development of innovative PD treatment strategies is imperative.

In the process of repairing nerve damage and treating neurodegenerative diseases, microglia play a unique and crucial role. Microglia, which are immune cells in the central nervous system, are considered to be macrophages in the brain and one of the first responders to central nervous system injury. Following a brain injury, microglia become activated and undergo a rapid transformation from a resting to an active state [2]. Activated microglia secrete numerous cytokines, chemokines, growth factors, reactive oxygen species, proteases, excitatory amino acids, and more, which can impact the survival of neurons [3]. Due to the complexity of their products, activated microglia are a “double-edged sword” in neurodegenerative illnesses. Similar to peripheral macrophages, activated microglia can be categorized as either “bad” M1 pro-inflammatory (classical activation) or “good” M2 anti-inflammatory and repair (selective activation) types based on their released cytokines and functional status [4]. So, modulating microglial polarization from M1 to M2 has been widely recognized as an effective strategy for the treatment of Parkinson’s disease (PD) [5].

“Metabolic reprogramming” describes the alterations in cellular metabolic processes that occur in response to changes in the external microenvironment, exhibiting distinct metabolic traits to furnish energy and essential biological compounds. In tumor cells, a preference for generating energy through glycolysis is observed even under oxygen-rich conditions, accompanied by suppressed oxidative phosphorylation. This phenomenon is commonly referred to as the Warburg effect [6]. The reprogramming of glucose metabolism serves to fuel the rapid proliferation of tumor cells and the synthesis of other biomolecules, thereby conferring a growth advantage. Disrupting this metabolic reprogramming in tumors has been shown to exert anti-tumor effects [7,8]. Recent studies have revealed the presence of the Warburg effect in M1 pro-inflammatory microglia, macrophages, and other immune cells. Although glycolysis is less efficient than oxidative phosphorylation in ATP production, this shift enables M1-activated microglia to generate ATP more rapidly. This rapid ATP production meets the acute energy demands of cells during inflammatory responses and facilitates the production of cytokines and reactive oxygen species. In contrast, resting microglia and those exhibiting an M2 anti-inflammatory repair phenotype rely on oxidative phosphorylation to provide a substantial and sustained supply of energy for their normal physiological functions [9]. In the lipopolysaccharide (LPS)-induced acute lung injury mouse model, mouse lung tissue and abdominal macrophages demonstrated heightened glycolysis and decreased levels of oxidative phosphorylation. Administration of the glycolysis inhibitor 2-Deoxy-D-glucose (2-DG) or blockade of the mTOR/HIF-1α glycolytic pathway hindered macrophage NLRP3 activation, ultimately curbing inflammatory factor production and alleviating symptoms in mice with acute lung injury [10].

Angelica sinensis, a traditional Chinese medicine, is widely utilized in clinical settings for its neuroprotective properties [11]. Levistilide A (LA, CAS No. 88182-33-6) is a characteristic phthalide extracted from Angelica sinensis [12]. It has been observed that LA can hinder endothelial cell activation and the expression of inflammatory cytokines, as well as reduce NLRP3 expression in human umbilical vein endothelial cells and vasculitis rat vascular tissue [13]. A recent study has shown that LA can reduce neuroinflammation by inhibiting the JAK2/STAT3 signaling pathway, play a role in neuroprotection and improve cognitive impairment in Alzheimer’s disease mice [14]. To our knowledge, the effect of LA on microglial glycometabolic reprogramming and Parkinson’s disease remains unexplored.

The main pathological feature of Parkinson’s disease is that the death of dopaminergic neurons leads to the denervation of the nigrostriatal pathway, resulting in the decrease in dopamine in the striatum [15]. Because the 1-methyl-4-phenyl-1, 2, 3, 6-tetrahydropyridine (MPTP), which is able to cross the blood-brain barrier, can induce the degeneration of dopaminergic neurons, the MPTP-induced mouse Parkinson’s disease model is commonly used for understanding the molecular mechanism of the disease and developing neuroprotective medicines [16]. Moreover, this model is easy to operate and cheap. This study aims to investigate the potential effects of LA in mouse models of Parkinson’s disease induced by MPTP and LPS-stimulated microglia. Our findings indicate that LA can regulate glucose metabolism, modulate inflammatory responses, and exert neuroprotective effects.

## 2. Results

### 2.1. LA Inhibits the Production of Inflammatory Cytokines NO, IL-6 and TNF-α in LPS-Induced Microglia

**Figure 1:**
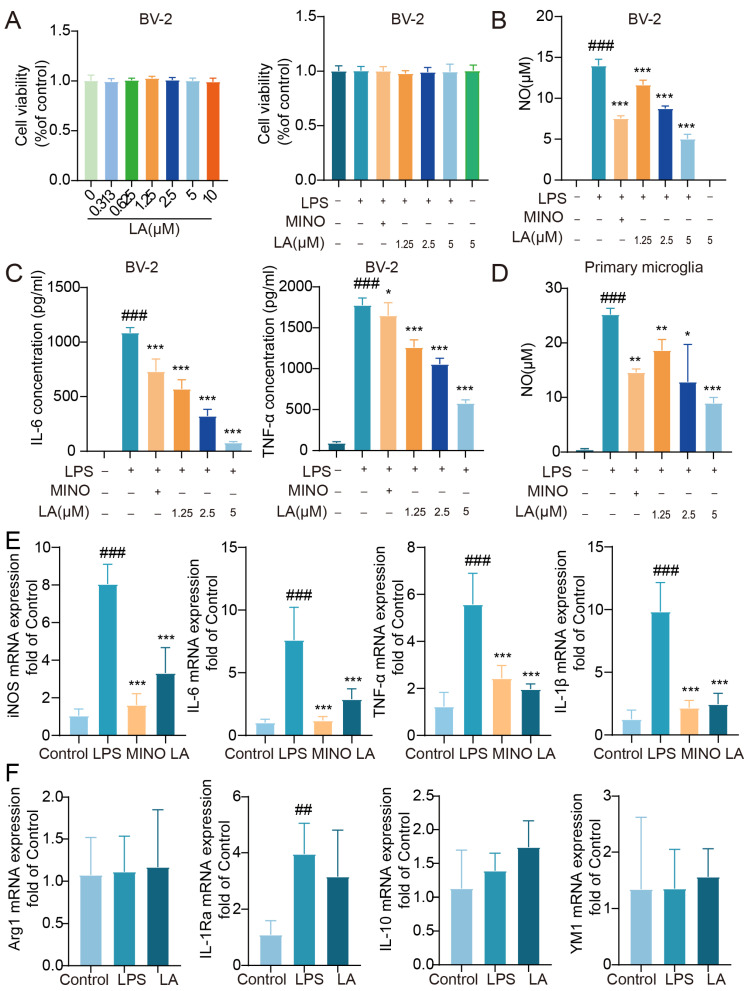
LA can inhibit the production of inflammatory cytokines NO, IL-6 and TNF-α in LPS-induced microglia. (**A**) LPS and/or LA had no effect on cell viability after LPS stimulation of BV-2 cells for 24 h, *n* = 6; (**B**,**C**) LA was able to inhibit LPS-induced NO, IL-6, and TNF-α production in BV-2 cells, *n* = 5; (**D**) LA was able to inhibit LPS-induced NO production in primary microglial cells. (**E**) LA inhibited the expression of proinflammatory cytokines mRNA in LPS-induced microglia; (**F**) LA does not inhibit anti-inflammatory factor mRNA expression vs. CON, ^###^ *p* < 0.001,^##^ *p* < 0.01; vs. LPS, * *p* < 0.05, ** *p* < 0.01, *** *p* < 0.001. Data are presented as the mean ± SD. LA: Levistilide A, LPS: lipopolysaccharide, MINO: minocycline.

We initially aimed to investigate whether LA has the potential to inhibit the production of inflammatory cytokines in LPS-stimulated BV-2 cells. LA had no significant impact on BV-2 cell viability at concentrations ranging from 0 to 10 μM, whether in the presence or absence of LPS (*p* > 0.05, Figure 1A). After stimulating BV-2 cells with LPS for 24 h, the levels of pro-inflammatory cytokines, including NO, IL-6, and TNF-α, were significantly higher in the LPS group compared to the Control group (*p* < 0.001). The positive control drug, minocycline, significantly inhibited the production of these cytokines. LA also inhibited the production of these cytokines in a dose-dependent manner (Figure 1B,C, *p* < 0.05). Similar results were observed in primary microglial cells (Figure 1D), consistent with those observed in the BV-2 cell line.

To further confirm the impact of LA on LPS-induced inflammatory factors in microglia, we utilized real-time quantitative PCR to assess the expression of pro-inflammatory factors, including iNOS, IL-6, TNF-α, and IL-1β mRNA. The results (Figure 1E) revealed that the expression of these pro-inflammatory factors induced by LPS was significantly reduced following treatment with the positive control drug minocycline or LA (*p* < 0.001). However, when compared to the LPS group, LA did not effectively inhibit the expression of the M2 anti-inflammatory factors Arg1, IL-1Ra, IL-10, and YM1 mRNA (*p* > 0.05, Figure 1F).

### 2.2. LA Inhibits LPS-Induced ROS Production in Microglia

**Figure 2:**
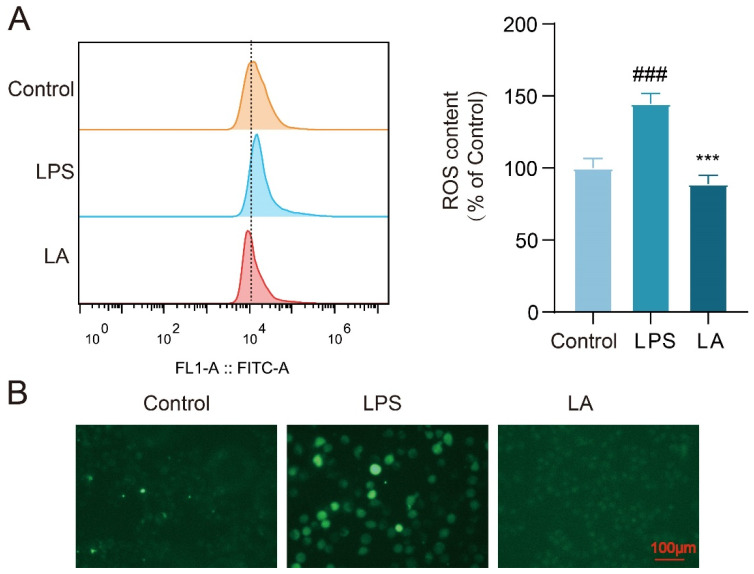
LA can inhibit LPS-induced ROS production in BV-2 cells. (**A**) Intracellular ROS content was detected by flow cytometry after 24 h of LPS-induced BV-2 cells; (**B**) Fluorescence microscopy images of BV-2 cells treated with LPS and/or LA for 24 h after incubation with the DCFH probe; vs. CON, ^###^ *p* < 0.001; vs. LPS, *** *p* < 0.001. Data are presented as the mean ± SD. LA: Levistilide A, LPS: lipopolysaccharide.

Upon microglia activation, a significant increase in reactive oxygen species (ROS) production occurs within the cells. Elevated levels of ROS have been associated with inflammation and neuronal cell death [17]. To evaluate the intracellular ROS levels, we utilized the fluorescent probe DCFH-DA. Flow cytometry results (Figure 2A) revealed that the intracellular ROS level was significantly higher in LPS-stimulated BV-2 cells after 24 h compared to the Control group (*p* < 0.001). However, following LA treatment, the ROS level in the LA group was significantly reduced (*p* < 0.001). Fluorescence microscopy results supported these findings (Figure 2B).

### 2.3. LA Inhibits Phosphorylation of IκB-α and Nuclear Translocation of NF-κB p65

**Figure 3:**
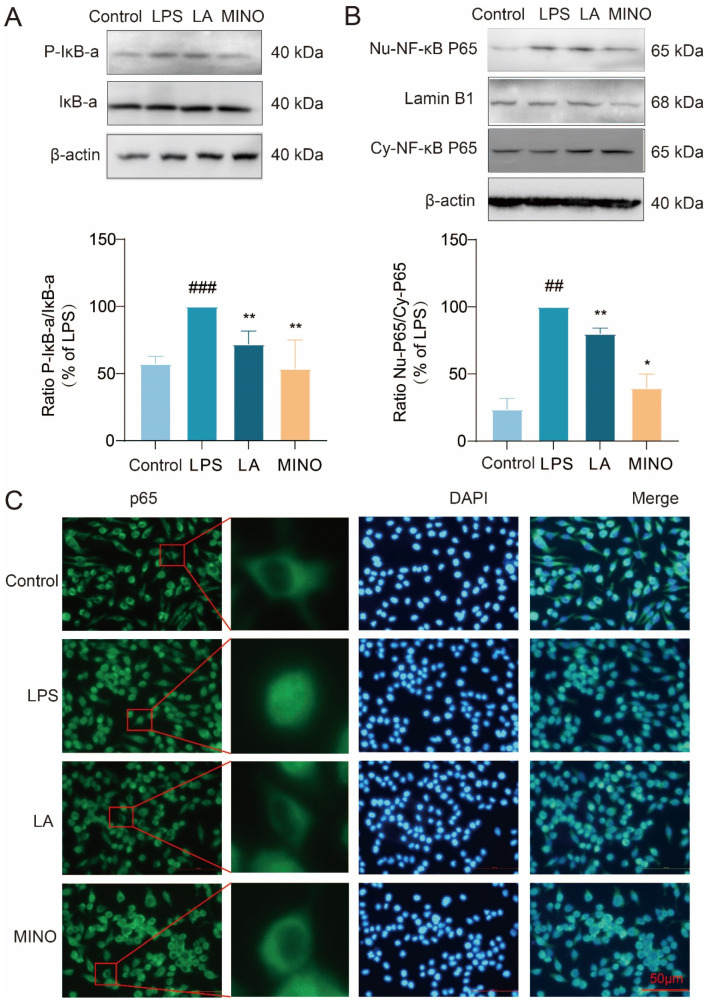
LA inhibits IκB-α phosphorylation and nuclear translocation of NF-κB p65. BV-2 cells were treated with LPS and or LA for 1 h; (**A**) Western blot results showed that LA was able to inhibit IκB-α phosphorylation; (**B**,**C**) The results of Western blot and fluorescence microscopy showed that LA was able to inhibit nuclear translocation of NF-κB p65; vs. Control, ^###^ *p* < 0.001, ^##^ *p* < 0.01; vs. LPS, * *p* < 0.05, ** *p* < 0.01. Data are presented as the mean ± SD. LA: Levistilide A, LPS: lipopolysaccharide, MINO: minocycline.

To investigate whether LA can hinder the nuclear translocation of NF-κB p65 and the phosphorylation of IκB-α, we utilized cellular immunofluorescence techniques and Western blot analysis. The Western blot results (Figure 3A,B) revealed that, following LPS stimulation of BV-2 cells for 1 h, LPS increased the phosphorylation level of IκB-α and caused the p65 subunit of NF-κB to translocate from the cytoplasm to the nucleus. Notably, LA hindered this translocation process. These findings were further validated by fluorescence microscopy images (Figure 3C). Collectively, these results suggest that LA can inhibit the activation of NF-κB transcription factors.

### 2.4. LA Inhibits LPS-Induced Reprogramming of Microglia Glucose Metabolism

**Figure 4:**
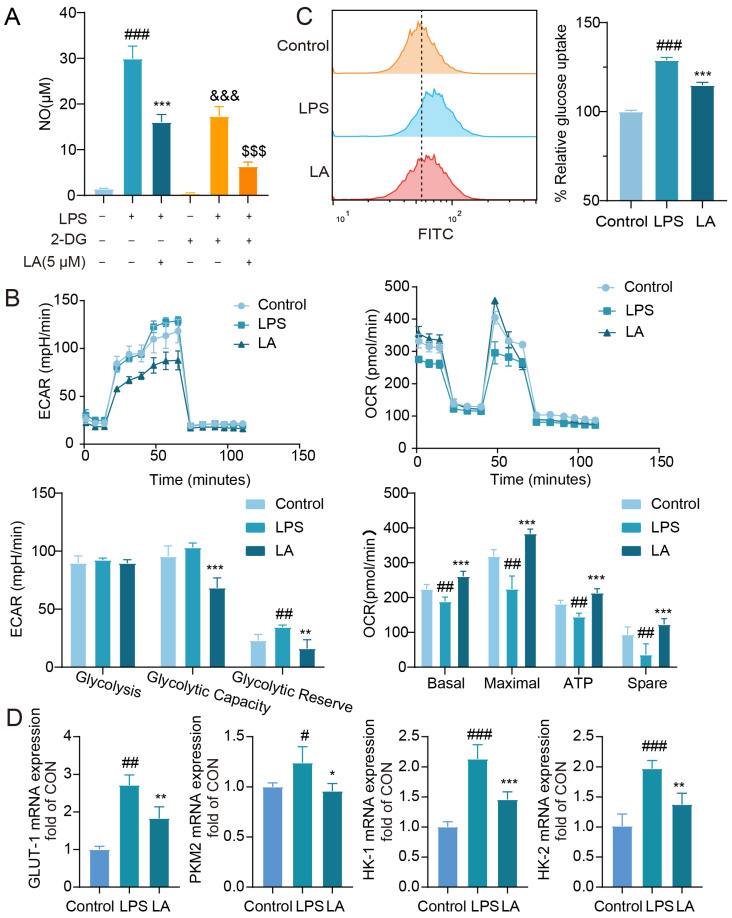
LA inhibits LPS-induced reprogramming of microglia glucose metabolism. (**A**) Effect of 2-DG on LPS-induced NO in BV-2 cell; (**B**) LA decreased ECAR and increased OCR in BV-2 cells (*n* = 4); (**C**) LA reduced glucose uptake in inflammatory microglia, *n* = 4; (**D**) LA reduced the mRNA expression of key glycolytic enzymes, *n* = 6; vs. CON, ^###^ *p* < 0.001, ^##^ *p* < 0.01, ^#^ *p* < 0.05; vs. LPS, * *p* < 0.05, ** *p* < 0.01, *** *p* < 0.001, ^&&&^ *p* < 0.001; vs. LPS+2DG, ^$$$^ *p* < 0.001. Mean ± SD, *n* = 6. Data are presented as the mean ± SD. LA: Levistilide A, LPS: lipopolysaccharide.

2-Deoxyglucose (2-DG) is a glycolysis inhibitor that has been reported to reduce inflammatory responses by suppressing glycolysis levels in macrophages [18]. To further explore the connection between glycolysis and microglia-mediated neuroinflammation, we evaluated the impact of 2-DG on LPS-induced NO release in BV-2 cells. As demonstrated in Figure 4A, treatment with 2-DG (2 mM) effectively inhibited LPS-induced NO release, indicating that modulation of glycolytic reprogramming reversed the M1 polarization state of microglial cells. Notably, when combined with LA, the co-treatment led to a more significant suppression of NO production compared to the LA group alone.

M1 and M2 microglia exhibit distinct metabolic patterns, with M1 microglia exhibiting elevated glycolysis and reduced oxidative phosphorylation levels, while M2 microglia display the opposite pattern with reduced glycolysis and increased oxidative phosphorylation [19]. Glucose is converted to pyruvate in the cytosol, which is further metabolized into lactate, leading to extracellular acidification. Elevated glycolysis results in lactate accumulation [20]. To investigate the impact of LA on glycolytic reprogramming, we utilized the Seahorse XF 96 Energy Metabolism Assay System. The extracellular acidification rate (ECAR) serves as a proxy for glycolysis levels. As shown in Figure 4B, LPS-stimulated microglia exhibited elevated glycolytic capacity and reserve, consistent with previous studies [21]. Notably, LA treatment reduced these levels, indicating that LA can mitigate glycolysis in microglia.

The Real-time Oxygen Consumption Rate (OCR) serves as a metric for mitochondrial function and represents the level of oxidative phosphorylation [22]. As demonstrated in Figure 4B, LPS-stimulated microglia exhibited reduced OCR values, indicating a decrease in oxidative phosphorylation. Notably, LA treatment reversed these effects, indicating that LA enhances the level of oxidative phosphorylation.

Studies have revealed that as cells’ glycolysis capacity increases, so does their glucose uptake [23]. Flow cytometry results (Figure 4C) indicate that LA treatment effectively inhibited the LPS-induced increase in glucose uptake (*p* < 0.001).

Subsequently, we utilized real-time quantitative PCR to assess the expression of key glycolysis enzymes. As displayed in Figure 4D, LPS stimulation upregulated the mRNA expression of glycolysis enzymes such as GLUT-1, PKM2, HK-1, and HK-2 in BV-2 cells. However, LA treatment significantly downregulated the expression of these enzymes.

### 2.5. Targeted Metabolomic Analysis of LA on LPS-Stimulated BV-2 Cells

Targeted metabolomics is to select some specific endogenous metabolites for accurate quantitative analysis with high sensitivity and accuracy. We applied a targeted metabolomics approach to detect changes in 19 glucose metabolism-related metabolites in BV-2 microglia.

**Figure 5:**
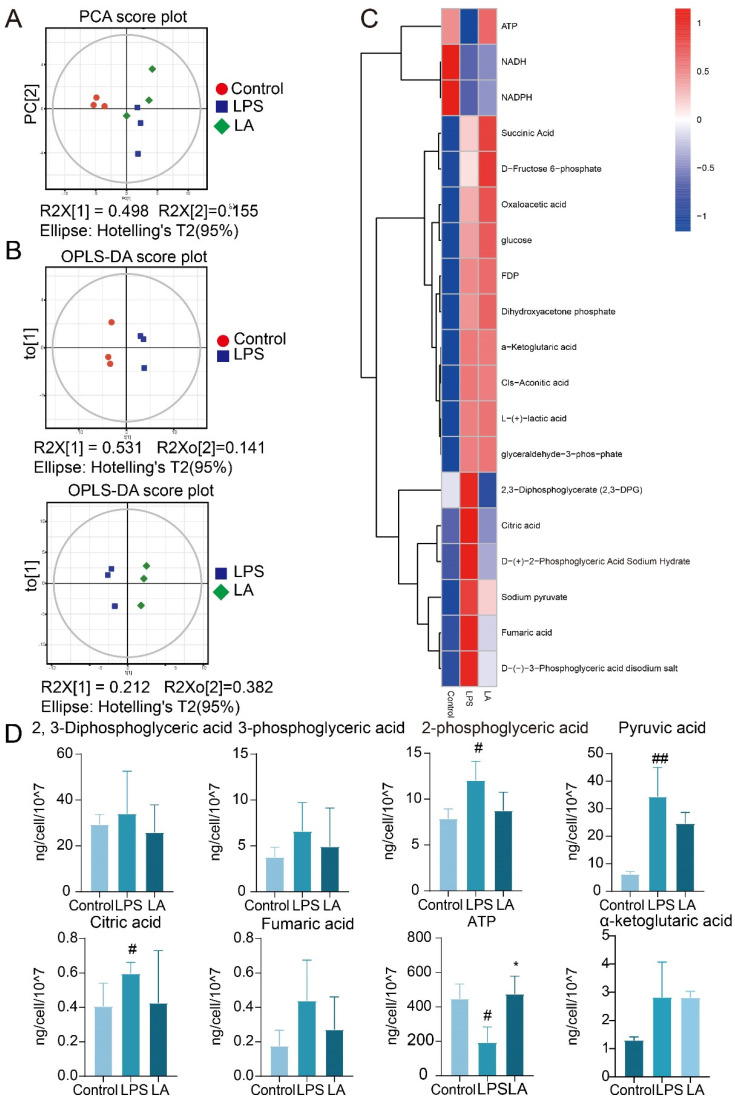
Central carbon target metabolome analysis. (**A**) PCA scatter plot of total sample of con group, LPS group and LA group; (**B**) OPLS-DA model scatter plot of CON group vs. LPS group and LPS group vs. LA group; (**C**) Hierarchical cluster analysis thermodynamic diagram; (**D**) Histogram of differential metabolites vs. CON, ^##^ *p* < 0.01, ^#^ *p* < 0.05; vs. LPS, * *p* < 0.05. Data are presented as the mean ± SD. LA: Levistilide A, LPS: lipopolysaccharide.

Principal component analysis (PCA) as well as orthogonal projections to latent structures-discriminant analysis (OPLS-DA) methods were used to analyze metabolites among different groups of BV-2 cells. As shown in Figure 5A,B, there were significant differences in metabolites between control, LPS and LA groups.

Figure 5C,D, show that after LPS stimulation of BV-2 cells, the contents of 2, 3-diphosphoglyceric acid, 3-phosphoglyceric acid, 2-phosphoglyceric acid, pyruvate, citrate, and fenugreek, tended to increase. This result was consistent with previous reports [24,25]. After the administration of LA, the levels of these metabolites were observed to decrease. When LPS stimulated BV-2 cells, glycolysis increased, oxidative phosphorylation decreased, and ATP levels subsequently decreased, aligning with previous reports [26]. However, LA treatment reversed these effects, elevating ATP levels. These findings suggest that LA can inhibit glycolysis product production while promoting oxidative phosphorylation product synthesis.

### 2.6. LA’s Impact on the AMPK/mTOR Signaling Pathway

AMP-activated protein kinase (AMPK) serves as a crucial energy sensor in eukaryotic organisms, regulating cellular energy metabolism. mTOR, a downstream target of AMPK, is widely distributed in the cytoplasm of living organisms. AMPK activation leads to the inhibition of mTOR phosphorylation, both of which play a role in the regulation of cellular metabolic processes.

**Figure 6:**
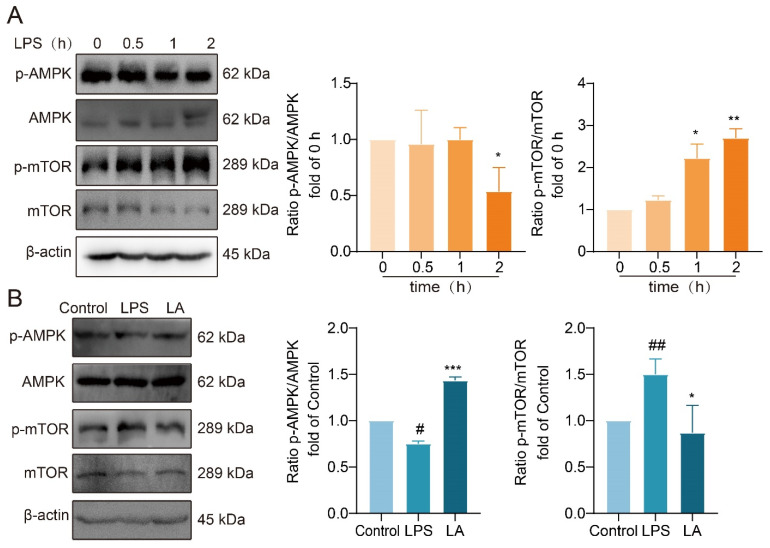
LA increases the phosphorylation level of AMPK and decreases the phosphorylation level of mTOR. (**A**) Effects of LPS on AMPK/mTOR pathway in BV-2 cells at 0, 0.5, 1, 2 h after LPS treatment. (**B**) Effect of LA on AMPK/mTOR in BV-2 cells induced by LPS for 2 h. vs. CON, ^##^ *p* < 0.01, ^#^ *p* < 0.05; vs. LPS, * *p* < 0.05, ** *p* < 0.01, *** *p* < 0.001. Data are presented as the mean ± SD. LA: Levistilide A, LPS: lipopolysaccharide.

Western blot analysis revealed that following 2 h of LPS stimulation, AMPK phosphorylation was significantly reduced, while mTOR phosphorylation was significantly increased (Figure 6A). However, LA treatment reversed these effects, significantly increasing AMPK phosphorylation (Figure 6B, *p* < 0.001) and decreasing mTOR phosphorylation (Figure 6B, *p* < 0.05) compared to the LPS group. These findings suggested that LA has a regulatory effect on the AMPK/mTOR signaling pathway, potentially influencing cellular metabolism and energy balance.

### 2.7. The Conditioned Medium from LA-Treated Microglia Protects Neurons

**Figure 7:**
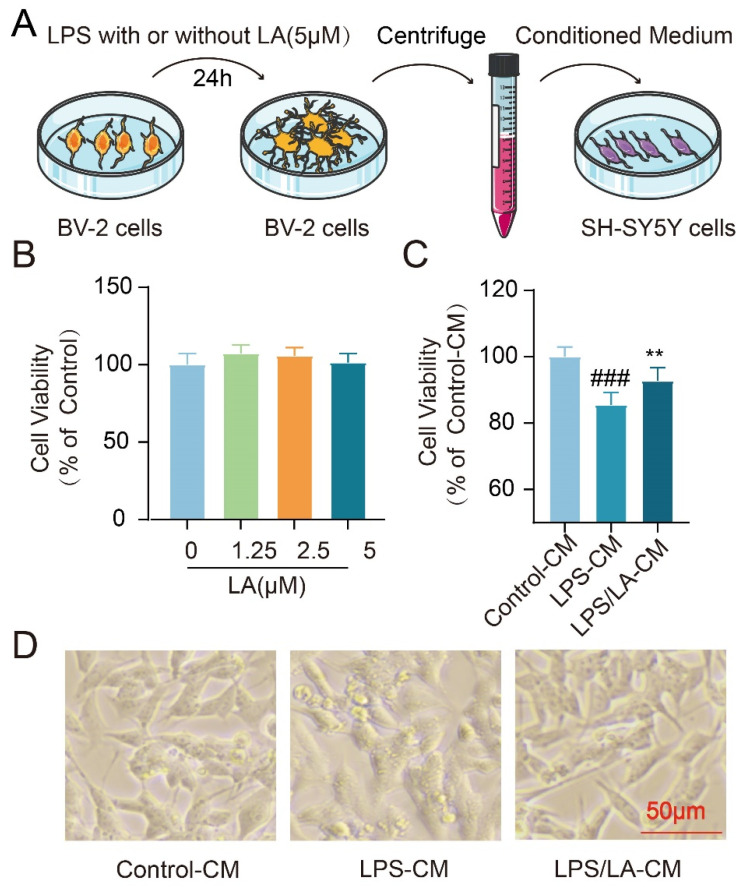
The Conditioned Medium from LA-treated microglia protects neurons. (**A**) Experimental process; (**B**) No effect of LA treatment alone on SH-SY5Y cell viability; (**C**) Microglia-conditioned medium co-treated with LPS and LA had a significant neuroprotective effect on SH-SY5Y; (**D**) Morphology of different conditioned culture treatments of SH-SY5Y cells. vs. CON, ^###^ *p* < 0.01; vs. LPS, ** *p* < 0.01. Data are presented as the mean ± SD. LA: Levistilide A, LPS: lipopolysaccharide.

Inflammatory cytokines secreted by activated microglia have the potential to induce neuronal cell death. In our previous study, we observed that LA suppressed the LPS-induced production of these pro-inflammatory factors. This raised the question: Could this inhibitory effect of LA exert neuroprotective benefits? To address this, we examined the impact of the conditioned medium from BV-2 cells on the survival of neuroblastoma SH-SY5Y cells (Figure 7A). Notably, LA alone, at concentrations ranging from 0–5 μM, did not affect the viability of SH-SY5Y cells (Figure 7B). However, when compared to the conditioned medium from the LPS-stimulated group, the medium derived from microglia treated with both LPS and LA demonstrated a remarkable neuroprotective effect on SH-SY5Y cells (*p* < 0.01, Figure 7C,D). These findings suggest that LA may confer neuroprotection by modulating microglia polarization.

### 2.8. LA Protects Dopaminergic Neurons and Inhibits Activation of Microglia in MPTP-Induced Parkinson Mice

**Figure 8:**
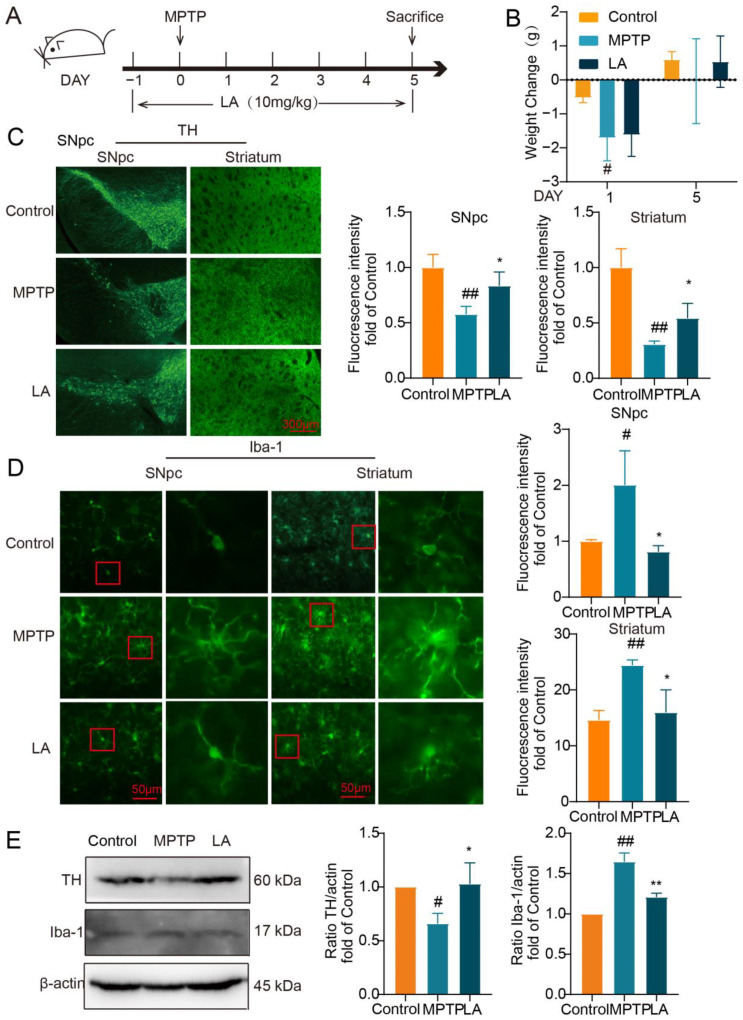
LA ameliorates dopaminergic neuronal damage and inhibits microglia activation in the brain of Parkinson’s disease mice. (**A**) In vivo experimental procedure; (**B**) Changes in body weights of mice; (**C**) Effect of LA on TH in the substantia nigra and striatum of Parkinson’s model mice; (**D**) Effect of LA on Iba-1 in the substantia nigra and striatum of Parkinson’s model mice. In the red squares are the microglia, which are magnified; (**E**) Western blot of TH and Iba-1 expression in the substantia nigra of Parkinson’s model mice. vs. CON, ^#^ *p <* 0.05, ^##^ *p* < 0.01, vs. MPTP, * *p <* 0.05, ** *p* < 0.01. Data are presented as the mean ± SD. LA: Levistilide A, LPS: lipopolysaccharide.

Subsequently, we utilized the MPTP-induced mouse model of Parkinson’s disease to assess the neuroprotective properties of LA in vivo (Figure 8A). As shown in Figure 8B, on the first day following MPTP administration, mice in the MPTP group exhibited a significant reduction in body weight (*p* < 0.05). After 5 days, the body weight of the MPTP group and LA group had recovered.

To evaluate the impact of LA on dopaminergic neuronal damage and microglia activation in the MPTP-induced mouse model of PD, we examined the expression of tyrosine hydroxylase (TH), a specific marker for dopaminergic neurons, and ionized calcium-binding adaptor molecule 1 (Iba-1), a sensitive marker for microglial activation. As demonstrated in Figure 8C, TH expression was significantly reduced in the substantia nigra and striatum of Parkinson’s mice (*p* < 0.05), while Iba-1 expression was elevated (Figure 8D, *p* < 0.05). Administration of LA reversed these effects, leading to increased TH expression (*p* < 0.05) and decreased Iba-1 expression (*p* < 0.01) in these brain regions. Western blot analysis further confirmed these findings (Figure 8E).

## 3. Discussion

The research has shown that LA can control ROS levels and inhibit tumor cell growth, ultimately leading to tumor cell death [27,28]. Additionally, LA has been found to improve memory deficits and cognitive decline in APP/PS1 transgenic mice with Alzheimer’s disease by inhibiting the production of inflammatory factors and reducing the deposition of beta-amyloid protein [29]. Furthermore, Angelica-derived LA has been reported to protect nerves and enhance the synthesis of the growth factor NGF [30]. These studies collectively demonstrate the pharmacological properties of LA, including its anti-tumor, anti-inflammatory, neuroprotective, and other beneficial effects. However, the impact of LA on activated microglia and PD remains unexplored.

PD is a complex neurodegenerative disorder associated with a depletion of dopamine [31]. Currently, levodopa, dopamine agonists, and inhibitors of dopamine-degrading enzymes are commonly used as symptomatic treatments for PD. Nevertheless, these therapeutic strategies possess several limitations, including a limited long-term efficacy and an inability to halt or reverse the progression of the disease [32].

Microglia, in their physiological state, play a crucial role in maintaining the brain’s internal environment, development, and cognitive functions [33]. A remarkable feature of microglia is their sensitivity to external stimuli, as changes in various microenvironmental factors can prompt rapid activation [34]. Activated microglia can be categorized as either M1 pro-inflammatory or M2 anti-inflammatory types. While this classification may simplify the diversity of microglia, it remains a valid concept, and numerous studies have employed this classification to investigate new therapeutic strategies. M1 microglia are capable of releasing a large number of damaging pro-inflammatory mediators, such as IL-1β, TNF-α, IL-6, and NO, accompanied by mitochondrial dysfunction, which promotes cellular oxidative stress, elevates the level of ROS, and directly contributes to neuronal apoptosis and exacerbates local inflammation [35,36]. On the other hand, M2 microglia display an anti-inflammatory and reparative phenotype, producing anti-inflammatory, trophic, and tissue-repair-promoting factors [37]. They also remove cellular debris and phagocytose neutrophils entering the brain, protecting neurons and promoting the migration of neural stem cells to ischemic zones to mitigate brain damage [38]. In the pathology of PD, microglia-mediated neuroinflammation plays a pivotal role and is a primary mechanism leading to the death of dopaminergic neurons. Therefore, the inhibition of microglia activation represents a potential therapeutic approach for PD, aiming to reduce dopaminergic neuron death [39]. In our study, notably, LA has been shown to inhibit the release of inflammatory cytokines, ROS production, and NF-κB p65 nuclear transfer, exhibiting neuroprotective effects both in vivo and in vitro. Interestingly, LA does not hinder the expression of anti-inflammatory cytokines.

Recent research has demonstrated the crucial role of metabolic reprogramming in the phenotypic transformation of microglia. In M1 microglia, the metabolic mode shifts from oxidative phosphorylation to glycolysis. Although glycolysis does not generate as much ATP as oxidative phosphorylation, it produces ATP rapidly, meeting the heightened metabolic demands of microglial cell growth during inflammatory states, as well as supporting the production of cytokines and ROS. This rapid ATP production allows M1 microglia to effectively respond to inflammatory challenges and propagate the immune response [40]. In a mouse model of perioperative neurocognitive impairment, it was observed that the hippocampus of animals in the surgical group exhibited altered microglial activation and an upregulation of M1 markers. Furthermore, surgical trauma was associated with a metabolic shift from oxidative phosphorylation to glycolysis in the hippocampus. In contrast, the pharmacological inhibition of glycolysis using 2-DG effectively mitigated the microglia M1 phenotype and the expression of pro-inflammatory mediators. This intervention also improved hippocampus-dependent cognitive functions, indicating a potential therapeutic approach for perioperative neurocognitive impairment [41].

Our findings demonstrate that the glycolysis inhibitor 2-DG effectively inhibits LPS-induced production of the inflammatory factor NO, aligning with previous reports [18]. The NO inhibition was even more pronounced after the administration of LA. OCR and ECAR, which are commonly used to represent the cellular capacity for oxidative phosphorylation and glycolysis, were significantly elevated in the LPS-activated microglia, consistent with the literature [42], while LA has the potential to reverse the metabolic mode shift observed in LPS-activated microglia. GLUT-1, PKM2 and hexokinase are key enzymes in glycolysis and play important roles in glucose transport, pyruvate production and mitochondrial stabilization, respectively [43]. Our findings indicate that LA significantly reduces the glucose uptake rate in LPS-induced microglia, accompanied by a reduction in glycolytic metabolite levels and a downregulation of key glycolysis enzyme genes. In summary, LA may promote a shift in the metabolic state of M1 microglial cells from glycolysis to oxidative phosphorylation.

The mTOR protein, serving as the central component of the cellular energy-sensing system, is responsible for facilitating the glucose metabolism pathway. AMPK, the primary sensor of energy status and regulator of metabolism in eukaryotic cells, phosphorylates tuberous sclerosis complex-2 (TSC2) and enhances its GAP activity on the small G protein Rheb (Ras homolog enriched in the brain), ultimately inhibiting mTOR phosphorylation [44]. We discovered that LA enhances the phosphorylation of AMPK and suppresses the phosphorylation of mTOR, indicating that LA reverses microglia metabolic reprogramming and inhibits their M1 phenotypic transformation through the AMPK/mTOR pathway.

To the best of our knowledge, no traditional Chinese medicine components have been reported to alter the polarization state of microglia by modulating metabolic reprogramming and providing a neuroprotective effect. This study fills this gap by demonstrating that LA, a phthalide-like constituent isolated from Angelica sinensis, can effectively suppress the phenotypic transformation of M1 microglia triggered by LPS. It also regulates the reprogramming of microglial glucose metabolism in an inflammatory state, potentially via the modulation of the AMPK/mTOR pathway. Furthermore, LA exhibits neuroprotective effects in an MPTP-induced Parkinson’s disease model. These findings provide valuable insights and data for future research into novel drugs for Parkinson’s disease and other neurodegenerative disorders.

## 4. Experimental Procedures

### 4.1. Animals

Twenty-two SPF male C57BL/6N mice, all 8 weeks old, were acquired from Beijing Charles River Biotechnology Co., Ltd. (Beijing, China). The animals were housed in an environment with a controlled temperature of 24 ± 2 °C and a relative humidity of 35 ± 5%. They were provided with free access to food and water and were allowed a week of acclimation before the administration of MPTP. All experiments were approved by the Animal Care and Use Committee at Tianjin University of Traditional Chinese Medicine (Animal Research Ethics approval number: TCM-LAEC2022022).

### 4.2. Drug

LA (CAS No. 88182-33-6) was purchased from the Chinese National Institute for the Control of Pharmaceutical and Biological Products (Beijing, China). The purity was more than 98%, which was determined by HPLC.

### 4.3. Cell Culture

The BV2 murine microglial cell line was cultured in Dulbecco’s modified Eagle’s medium (DMEM, Gibco, Waltham, MA, USA) supplemented with 10% heat-inactivated fetal bovine serum (Gibco), 100 U/mL penicillin, and 100 μg/mL streptomycin (VivaCell, Shanghai, China). Primary microglia were derived from postnatal day 1–3 Wistar rat brains as described previously [45]. The entire forebrain was harvested from the rats after decapitation, digested with trypsin, and filtered through a 40 μm sieve to obtain a cell suspension. The cells were then cultured in T75 culture flasks for 14 days. After 14 days, the cells were purified on a shaker, and they could be used for subsequent experiments.

### 4.4. Cell Viability Assay, Detection of Nitric Oxide and Cytokines

The BV-2 microglia were seeded in 48-well plates at a density of 1.6 × 10^5^ cells per well, while the primary microglia were inoculated in 96-well plates at a density of 1 × 10^5^ cells per well. The microglia were pretreated with LA for 30 min before being treated with LPS and/or LA for 24 h. Cell viability was determined using the CCK-8 assay. The supernatant from the cell cultures was used to measure the levels of NO and inflammatory factors. NO was detected using the Griess method according to the manufacturer’s instructions (Beyotime, Shanghai, China, S0021S). TNF-α and IL-6 were measured using commercial Elisa kits (RD, St. Paul, MN, USA, MTA00B and M6000B).

### 4.5. RNA Isolation and Quantitative PCR

BV-2 cells were seeded in 6-well plates (8 × 10^5^ cells/Well). After treatment for 8 h (for inflammatory factors) or 24 h (for glycolysis-related enzymes), the mRNA expression was detected using quantitative PCR. RNA was extracted following the instructions (Progema, Madison, WI, USA, LS1040). The RNA quality was tested with A260/280. All the results were ≥2.0, indicating that the purity of RNA was qualified. Then, 200 ng of the resulting RNA was added to a 20 μL system for reverse transcription to cDNA. qPCRs were performed in a 10 µL reaction with 0.5 μL cDNA and SYBR Premix (ABI, Waltham, MA, USA, A25742), and the specific primer sequences were added (Table 1). Reaction conditions were 50 °C 2 min, 95 °C 2 min, (95 °C 15 s, 60 °C 1 min) × 40. Four sets of biological replicates and four sets of technical replicates were measured in parallel. RT negative control was also performed to monitor DNA contamination.

### 4.6. Flow Cytometry

BV-2 cells were seeded in 12-well plates (3 × 10^5^ cells/well). After 24 h of LPS and/or LA treatment, the cells were washed and collected. To detect ROS, cells were incubated with DCFH probe (10 μM) for 1 h in the dark. For the assessment of glucose uptake capacity, cells were treated with 2-NBDG (50 μM) for 1 h. After another PBS wash, the levels of ROS and glucose uptake capacity were analyzed by flow cytometry.

### 4.7. EACR and OCR

To conduct an analysis of extracellular acidification rate (ECAR) and oxygen consumption rate (OCR), BV-2 cells (1 × 10^4^ cells per well) were examined using an XF-96 Extracellular Flux Analyzer (Agilent, Santa Clara, CA, USA). The ECAR was evaluated in response to various stimuli, including 10 mM glucose, 1 µM oligomycin, and 50 mM 2DG (all from Agilent, 103020-100). Additionally, ECAR was analyzed in response to 1.5 µM oligomycin, 1 µM FCCP, and 0.5 µM rotenone/antimycin A, following the manufacturer’s instructions.

### 4.8. Targeted Metabolomics Analysis

BV-2 cells were seeded in 100 mm dishes overnight and stimulated by LA and/or LPS for 24 h. The cell precipitates were flash-frozen in liquid nitrogen. After the addition of a 50% acetonitrile water solution, the cells were sonicated for 30 min and centrifuged. Then, to the cell was added 150 μL of 3-NPH (200 mM) and 150 μL of EDC (120 mM; containing 6% pyridine), and the reaction proceeded for 1 h at 40 °C in a water bath. The supernatant was filtered through a 0.22 μm membrane and used for LC-MS/MS analysis. The mass spectrometry conditions were as follows: Ion source: ESI ion source; Curtain Gas: 35 arb; Collision gas: 7 arb; Ion spray voltage: 4500 V; IonSource Temperature: 450 °C; IonSource Gas1: 55 arb; IonSource Gas2: 55 arb.

### 4.9. The Effect of Conditioned Medium on Neurons

BV-2 cells were seeded in 48-well plates (1.6 × 10^5^ cells/well). After a 30 min drug pretreatment, LPS and/or LA were added to the BV-2 cells and incubated for 24 h. The supernatant of the culture medium was then collected. The culture medium of SH-SY5Y cells was replaced with the corresponding conditioned medium and incubated for another 24 h. Cell viability was then assessed using the CCK-8 method.

### 4.10. Western Blotting

Cells were seeded in 6-well plates (8 × 10^5^ cells/well). The total protein, cytoplasmic and nuclear proteins were extracted after the cells were treated with LPS and/or LA for 1 h or 2 h. Western blot analysis was then performed using appropriate primary antibodies and horseradish peroxidase (HRP)-conjugated secondary antibodies, along with the ECL chemiluminescence kit (Millipore, Darmstadt, Germany, WBKLS0100). All the primary antibodies were purchased from CST company: β-actin (3700S), NF-κB p65 (8242S), IκB-α (4812S), p-IκB-α (2859S), Lamin b1 (17416S), mTOR (29182S), AMPK (5831S), p-AMPK (2535S), TH (58844S), and Iba-1 (17198S).

### 4.11. MPTP-Induced Mouse Parkinson Model

Mice were randomly assigned to three groups: Control (*n* = 6), MPTP (*n* = 8), and LA (*n* = 8). To establish an acute MPTP mouse model, after injecting 250 mg/kg of probenecid sodium intraperitoneally, MPTP (22 mg/kg) was intraperitoneally administered for the first time 30 min later, and MPTP was injected every two hours for four times in one day [46,47]. LA dissolved in sterile corn oil was administered by intraperitoneal injection daily, starting the day before MPTP injection and continuing for 7 days, at a dose of 10 mg/kg once a day [14,48]. The conversion of human equivalent dose was 1.01 mg/kg. In the Control and MPTP groups, mice received sterile corn oil injections according to their body weight and administered intraperitoneally.

### 4.12. Immunohistochemistry

Seven days post-administration, mice were anesthetized with pentobarbital sodium and perfused. Brain tissues were harvested. The entire brain tissue was fixed in 4% paraformaldehyde and dehydrated in sucrose solution. Frozen sections of mouse brain tissue were prepared, and coronal sections with a thickness of 40 μm from the striatum to the substantia nigra were selected for immunofluorescence staining. Brain tissue sections were perforated with PBS containing 0.3% Triton X-100, then blocked. Rabbit primary antibodies (TH, Iba-1, CST, 1:100) were added and incubated at 4 °C overnight. After washing with PBS, goat anti-rabbit IgG-labeled fluorescent secondary antibody (1:1000) was added and incubated for 1 h at room temperature in the dark. Photographs were taken using a confocal fluorescence microscope with an excitation wavelength of 488 nm.

### 4.13. Statistical Analysis

All experimental data underwent tests for homogeneity of variance and normality. The data were presented as mean ± standard deviation and analyzed using SPSS version 26.0. The Shapiro-Wilk test was performed to check whether the data values were normally distributed, and variance homogeneity tests were used to detect homogeneity of variance. In the case of homogeneous variance, an independent sample *t* test was used to compare two groups of data, and a one-way analysis of variance LSD test was used to compare multiple groups of data. In the case of heterogeneous variance, a non-parametric test was adopted to compare two groups of data, and the TamhaneT2 test was adopted to compare multiple groups. A *p*-value less than 0.05 was considered statistically significant.

## Figures and Tables

**Table 1:** Primer sequences of quantitative PCR.

Gene	Primer Pair (5′-3′)	Accession ID
GAPDH	F: CTTCACCACCATGGAGAAGGC R: GGCATGGACTGTGGTCATGAG	XM_001476707.5
iNOS	F: GGCAGCCTGTGAGACCTTTG R: GCATTGGAAGTGAAGCGTTTC	XM_006532446.3
TNF-α	F: CGGGGTGATCGGTCCCCAAAG R: GGAGGGCGTTGGCGCGCTGG	NM_001278601.1
IL-6	F: CCAGAGATACAAAGAAATGATGG R: ACTCCAGAAGACCAGAGGAAA	NM_001314054.1
IL-1β	F: CGCAGCAGCACATCAACAAGAGC R: TGTCCTCATCCTGGAAGGTCCACG	XM_006498795.3
IL-1Ra	F: AAGCCTTCAGAATCTGGGATAC R: TCATCTCCAGACTTGGCACA	NM_001159562.1
Ym1	F:TCACTTACACACATGAGCAAGAC R: CGGTTCTGAGGAGTAGAGACCA	NM_009892.3
Arg1	F: GGAAGACAGCAGAGGAGGTG R: TATGGTTACCCTCCCGTTGA	NM_007482.3
IL-10	F: GCTCTTACTGACTGGCATGAG R: CGCAGCTCTAGGAGCATGTG	NM_010548.2
β-actin	F: AGAGGGAAATCGTGCGTGACATCAA R: ATACCCAAGAAGGAAGGCTGGAAAA	NM_007393.5
HK1	F: TGCCATGCGGCTCTCTGATG R: CTTGACGGAGGCCGTTGGGTT	NM_010438.3
PKM2	F: AGGATGCCGTGCTGAATG R: TAGAAGAGGGGCTCCAGAGG	NM_011099.4
HK2	F: TCATTGTTGGCACTGGAAGC R: TTGCCAGGGTTGAGAGAGAG	NM_013820.3
GLUT-1	F: CAGTTCGGCTATAACACTGGTG R: GCCCCCGACAGAGAAGATG	NM_011400.3

## Data Availability

Data are contained within the article.
